# 3D bioprint me: a socioethical view of bioprinting human organs and tissues

**DOI:** 10.1136/medethics-2015-103347

**Published:** 2017-03-20

**Authors:** Niki Vermeulen, Gill Haddow, Tirion Seymour, Alan Faulkner-Jones, Wenmiao Shu

**Affiliations:** 1 Department of Science, Technology and Innovation Studies, University of Edinburgh, Edinburgh, UK; 2 Department of Biomedical Engineering, University of Strathclyde, Glasgow, UK

**Keywords:** Animal Experimentation, Donation/Procurement of Organs/Tissues, Engineering, Stem Cell Research, Applied and Professional Ethics

## Abstract

In this article, we review the extant social science and ethical literature on three-dimensional (3D) bioprinting. 3D bioprinting has the potential to be a ‘game-changer’, printing human organs on demand, no longer necessitating the need for living or deceased human donation or animal transplantation. Although the technology is not yet at the level required to bioprint an entire organ, 3D bioprinting may have a variety of other mid-term and short-term benefits that also have positive ethical consequences, for example, creating alternatives to animal testing, filling a therapeutic need for minors and avoiding species boundary crossing. Despite a lack of current socioethical engagement with the consequences of the technology, we outline what we see as some preliminary practical, ethical and regulatory issues that need tackling. These relate to managing public expectations and the continuing reliance on technoscientific solutions to diseases that affect high-income countries. Avoiding prescribing a course of action for the way forward in terms of research agendas, we do briefly outline one possible ethical framework ‘Responsible Research Innovation’ as an oversight model should 3D bioprinting promises are ever realised. 3D bioprinting has a lot to offer in the course of time should it move beyond a conceptual therapy, but is an area that requires ethical oversight and regulation and debate, in the here and now. The purpose of this article is to begin that discussion.

## Background: ‘grow your own organs’

Recent media headlines suggest that scientists will in the future have the ability to create or ‘biofabricate’ personalised organs such as livers and hearts through a process known as three-dimensional (3D) bioprinting.^1–3^ This is the biological variant of the recent trend towards 3D printing; small-scale manufacturing of computer-designed forms through laying down successive layers of material until the entire object is created. This development draws on longstanding printing technology, which after the invention of the printing press in the 15th century made books, and thereby knowledge, widely available and affordable. Later in the 20th century with the development of the photocopier and inkjet printers, the hardware component was set. While 3D printing is working with inorganic materials, the intention of bioprinting is to work with organic materials (including living cells) to create structures approximating body parts. Specialised bioprinters use biological inks (bioinks—such as differentiated, human embryonic or induced pluripotent stem cells (iPSCs)) to print 3D constructs composed of living organic materials. These new forms of printing, should they be realised, will, it is argued, have the same revolutionary and democratising effect as book printing in their applicability to regenerative medicine and industry. Individually designed biological structures or body parts will become as available as text in modern literate societies.

There are obvious links drawn between 21st century 3D bioprinting and the development of the 15th century printing press in terms of process as well as similarities in the nascent development stages regarding the effect of resources and access for both printing technologies.

Long-term 3D bioprinting has the potential to be a ‘game-changer’, providing an alternative source of organs no longer necessitating the need for living or deceased human donation as human organs would be printed on demand. Nonetheless, there are key differences between printing and 3D bioprinting. The latter is distinctive in terms of the technological process of printing, the organic products that are involved, the therapeutic purpose and, finally, in terms of placing the printed organ within a human body. The technology is not yet at the level required to bioprint an entire organ. A realistic and short-term goal is for 3D bioprinting to create alternatives to animal testing. For example, drug testing can be accomplished via bioprinted structures embedded within lab-on-a-chip devices and even improved by the dramatic increase in throughput the technology enables.^4–6^ Furthermore, a mid-term gain still to be realised however, relates to the creation of tissue components such as human heart valves, especially for younger members of the population (eg, paediatric patients) who suffer specific problems with current bioprosthetic or mechanical heart valve (MHV) options. The required tissue components are created from the patient's own cells (thus reducing the risk of rejection) and the geometry (size and shape) of the components can be customised to match perfectly with the patient's requirements. Unlike mechanical implants, such engineered tissue components that are 3D bioprinted have the ability to grow with the patient, eliminating the need for further operations to replace components which are no longer suitable.^7^

**Figure 1:**
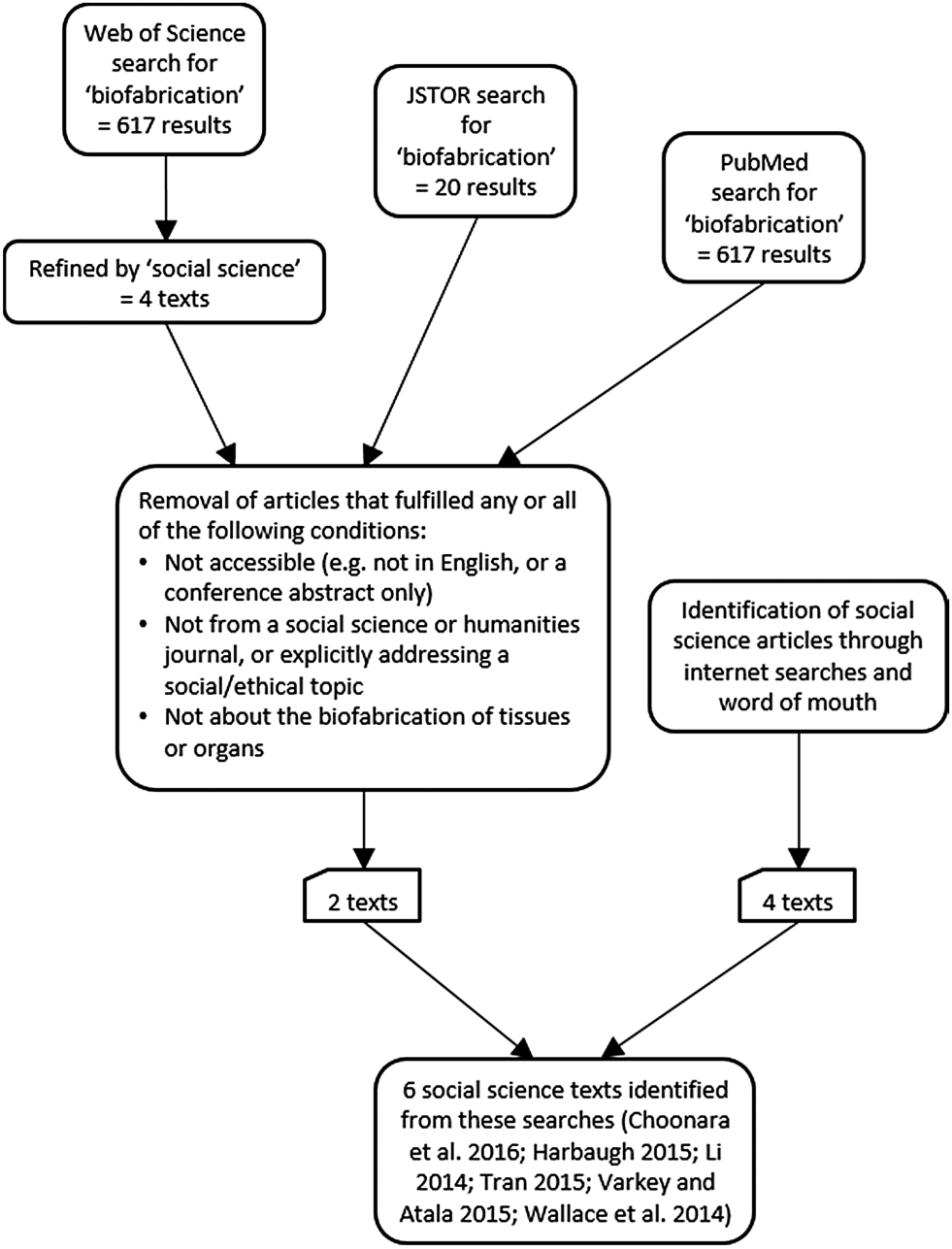
Literature search sources for social science literature on biofabrication undertaken in March–June 2016.

Given the promise of the technology to solve entrenched ethical issues relating to supply and demand of human or non-human animal transplants, a thorough review of any specific socioethical pitfalls is instructive. In order to scope the existing social science literature in the field of bioprinting, literature searches of articles and book references were undertaken on the databases PubMed, Web of Science and JSTOR in March and April 2016, and undertaken again in June 2016 in order to identify any extra articles (figure 1). In addition to this, the Institute of Physics journal *Biofabrication* was searched as a leading journal in this area of biofabrication and bioprinting. This review exercise found that with only a few notable exceptions from the areas of law or social sciences,^8–15^ the current literature on bioprinting specifically (rather than traditional 3D printing) is located entirely within the biological sciences and medical sciences. Much of the medical and scientific literature, however, does focus on some social aspect of the technology within discussion to some extent. This allows analysis of both what is already being highlighted within scientific articles, and allows the identification of areas where social science research might build on existing themes or fill gaps in understanding. The latter we will address through offering both positive and negative analysis of the socioethical challenges that might lie ahead.

Although bioprinting can avoid ethical dilemmas associated with xenotransplantation and clinical organ transplantation, it is not without its own challenges, practical, ethical and regulatory, which we will need to address. These relate to managing public expectations and the ethics of biomedicine's continuing reliance on costly technoscientific solutions. For example, the expense of creating your own personalised organ may mean that waiting lists are the equivalent of waiting on an allograft or human transplant.

As such, it seems important to explore possible scenarios of 3D bioprinting and its applications for tissue and organ creation. Hence, we hope to begin discussion with a modest contribution. After outlining extant literature and given we found little ethical or social commentary from an arts, social science and humanities perspective, we offer a comparative ethical analysis weighing up a few of the benefits and challenges 3D bioprinting may cause should its promise be realised. There are different frameworks for engaging with social science and ethical aspects of new technologies and we outline and discuss whether Responsible Research Innovation (RRI), currently favoured, may be a way to deal with and reflect on 3D bioprinting, especially around interdisciplinary ethics. 3D bioprinting may have a lot to offer in the course of time, but as an area, it needs ethical oversight and regulation as well as debate. A debate we hope to begin.

## 3D bioprinting and biofabrication

3D printing is undoubtedly proven successful and 3D printers are now widely used and commercially available. Bioprinting as a particular area regularly discussed in both academic literature and the media in connection alongside 3D printing techniques that use non-biological materials (eg, plastics, metals, fabrics or ceramics). 3D printing of objects has rapidly developed from being a niche and expensive development on two-dimensional (2D) printing technology in the 1980s to now being an extremely rapidly developing industry with ever-increasing applications.

Bioprinting is a far more complex matter, which is explored in the emerging field of biofabrication. Although the terms bioprinting and biofabrication are often used interchangeably and do overlap, the most up-to-date definition is offered which delineates between natural biomineralisation (eg, pearls) and technological biofabrication: bioprinting and bioassembly. The latter, used for tissue engineering and regenerative medicine, is described as the:

… the automated generation of biologically functional products with structural organization from living cells, bioactive molecules, biomaterials, cell aggregates such as micro-tissues, or hybrid cell-material constructs, through Bioprinting or Bioassembly and subsequent tissue maturation processes.^16^

And bioprinting as defined in Oxford dictionary:

The use of 3D printing technology with materials that incorporate viable living cells, for example, to produce tissue for reconstructive surgery.^17^

At the moment, biofabrication exhibits many elements of an emerging new community in science,^18^ including an annual conference, an international society for biofabrication, several journals and new educational programmes at universities around the world. Biofabrication and the subdivision of bioprinting connect closely to the large and multidisciplinary areas of tissue engineering and regenerative medicine in both their techniques (the use of biological materials to build constructs) and in the goal of using the body's own processes to replace and regenerate the body. Scientists trace back the start of bioprinting to over three decades ago, with the appearance of articles that started to explore the possibilities to organise cells spatially into structures that closely mimic the native tissue architecture and can potentially help to fabricate engineered tissue.^19–21^

A primer into bioprinting distinguishes different approaches, technologies and materials.^22^ First, different printing technologies are used, including inkjet, bioextrusion, laser-assisted bioprinting and valve-based techniques. Second, different forms of bioprinting are distinguishable: rational design and autonomous self-assembly. While biomimicry aims to manufacture identical reproductions of cellular and extracellular components of tissue or organs through rational design, autonomous self-assembly takes embryonic organ development as a guide, replicating tissue by relying on the capacity of cell(s) to generate functional and structural properties of tissue through self-organisation. Building on these two processes, organoids, the functional building blocks of tissues and organs, are constructed. For such tissue engineering, pluripotent stem cells (PSCs) are the favoured cells to use given their ability to both self-renew and differentiate into any required adult cell type^4^; this means that PSCs can be expanded into the large numbers required and then programmed to differentiate into the required organ-specific cell lines. Unlike primary cells, which have a limited number of expansion cycles, PSCs are divided into embryonic stem cells (ESCs) and iPSCs. The ethics of using ESCs remain controversial in some countries, especially those such as the USA where the use is heavily restricted. Although ESCs are quite commonly used, adults' own cells are reprogrammed into iPSCs for use. Ideally, the use of iPSCs can overcome any problems of an immunological response to the bioprinted organ. Induced-PSCs have not been inserted into a human being in this way and the risks of doing so currently incalculable. Moreover, in addition to the cells that are chosen to print with, it is crucial to pick the right medium in which to suspend the cells (of multiple different cell types) or scaffold material for the printing process, such as natural polymers or synthetic hydrogels to support bioprinting of 3D structures.

## Possible short-term benefits of 3D bioprinting

### Short-term benefits of avoiding animal testing

As previously mentioned, there are short-term goals that can be accomplished with the development of 3D bioprinting. Short-term applications could relate to drug testing (thereby reducing the need for animal testing) as testing of drugs could take place on ‘organ-on-a-chip’ technologies. A person's own cells can be reprogrammed into iPSCs, which can be used to generate bioprinted organoids that test the potency and efficacy of pharmaceutical drugs. Potentially, this offers a more ethical process than the use of animals for drug testing and a more efficient and reliable one as the testing is done on human tissues. Indeed, there is the further potential for personalised medicine, where a particular individual's reaction to a drug can be gauged alleviating the incidence of adverse drug reactions and dosages can be adjusted to suit the required efficacy.

### Short-term benefits of tissue engineering

Apart from the ‘personalised medicine’ opportunities offered by 3D bioprinting, robust technological advances are made in terms of the tissue engineering. Artificial skin, cartilage and tracheas have been tissue engineered,^23^ and there have been advances in work on the printing of bone,^24^ parts of the ear^11^ and of heart valves.^25^ Bladders have been ‘moulded’ and successfully implanted into a patient.^26^ However, all of these are relatively simple structures and such techniques are not easily (if at all) transferrable to engineering complex solid organs, which are more applicable for drug testing applications and more in demand for organ replacement^4^ (http://www.nhsbt.nhs.uk/).

### Short-term benefits of disease modelling

Another possibility exists if instead of healthy tissue were to be bioprinted, one could add cancerous tissue and disease models^27^ in order to study the efficacy of novel treatments outside the patient’s body. By incorporating the patient's own cells more accurate models and hence more effective treatments should result.

## Possible long-term benefits

### Long-term benefits: avoiding the animal question

Although yet to succeed, researchers are now working on the fabrication of solid 3D organs.^28^ ^29^ Regenerating human organs is potentially a game-changer in the volume and process of repairing and replacing human organs. Bioprinting may be more ethically robust than genetically altering and then growing organs in host animals. Chimaera pigs bring into question how such scientific objects can be ethically regulated when it is difficult to classify what is human or what is animal.^30^ Further rising attention is given to the status of non-human animals. The term ‘non-human’ animals refers to the increasing recognition that some animals share cognitive and emotional characteristics with humans. Public attitudes demonstrate that although participants suggested it was ‘morally acceptable’ to develop and research xenotransplantation,^31^ only 77% of the Swedish public were willing to accept an organ from a relative, 69% from a deceased person, 63% an artificial ‘organ’ and 40% an animal organ.^25^ ^32^ From empirical sociological research available, the mixing of animal and human materiality produces public reactions of disgust or ‘yuck’.^33–36^ Unlike xenotransplantation, which remains highly experimental despite advances using CRISPr technology to modify porcine hearts in order to prevent rejection, 3D printing organs circumvents intractable problems relating to source and process.

### Long-term benefits: avoiding the possibility of ‘yuck’

Shaw *et al* 37 have argued how chimaera pigs, used to incubate human organs created through the implantation of iPSCs, can be an ethical source of immunocompatible organs. While this is indeed a good parallel development, bioprinting seems an attractive alternative as it avoids the ‘yuck’ factor.^33^ ^36^ The descriptive basis of using the ‘yuck’ factor as originally discussed by Kass^38^ has been expanded as a normative one to ask questions such as whether the ‘yuck’ factor should be used as the basis for social and ethical debates or institutionally formalised.^39^ Our intention here is not to challenge the usefulness of the concept in ethical or legal debates but to state that, as a social reaction, it exists as an emotion regardless of whether or not it ought to be involved in moral judgements.^39^ The ‘yuck’ response is, as Mary Douglas argues, linked to ideas about ‘Pollution behaviour' the reaction which condemns any object or idea likely to confuse or contradict cherished classifications’ as out-of-place.^36^ ^40^ Hence, although ethicists and philosophers have argued that the normative power of ‘disgust’ is limited and can do no moral work, others have found it a powerful way to discuss how social attitudes are reacting to the way that technology is challenging what is presumed as natural:

The contemporary need for naturalness can be better understood as a response to the fact that technology makes reality more and more makeable and, consequently, more contingent. Advancing technology changes everything that is, into our object of choice…[I]f human nature itself becomes makeable, it can no longer naively be laid down as the norm.^41^

The Nuffield Council of Bioethics (2015)^42^ in a recent analysis of the role ‘natural’ plays in public debate argues that it is a term best be avoided because of the variability in its use over time. We suggest that 3D bioprinting avoids all debates about yuck and what is natural and hence is likely to be more ethically robust in terms of social acceptability than other areas of biotechnologies.

### Long-term benefits: end the financial costs in human organs

Evidence suggests that the human organ transplantation rates have plateaued and indeed is a victim of its own technological success; as a greater range of organs can be transplanted, more organs will be needed. And for organs that are not paired and cannot come from living donors, organs rest on an ethically double-edged sword; in order to save life another person has died. Organ regeneration would eliminate the need for human organs and therefore in the long run also reduce the medical costs associated with transplantation.^8^ It could also end the illegal trade in organs and human abuses that occur with it.^43^

### Long-term benefits: with immediate ‘youth’ value

Organs for young adults and children have always been difficult to procure for all the reasons stated above as well as having to come from a young donor. Some applications of 3D printing may in the future be primarily directed at a young age cohort given young people's suitability to receive a bioprinted replacement organ as their bodies can grow around with the organs.^44–46^ Equally, younger individuals who suffer from heart valve disease can have issues relating to using bioprosthetic heart tissue valves. Such valves from porcine sources only last as long as the pig donor would have done; approximately 10–15 years and therefore, the tendency is to use them with older patients. Using MHVs last longer but with the side effects of a lifetime on anticoagulants for younger recipients. The ‘appeal’ then of 3D bioprinting, or of ‘here is one made earlier’ is a much more attractive option than a lifetime on medication.

## Bioprinting—ethical challenges

There has been little discussion of risks in the social science or ethics literature that we have found; hence, this can lead to assumption that there are no risks. Moreover, knowledge of opinions, views and attitudes of publics and patients is essential in an area where little is understood. Therefore, we will use the rest of this article to outline ethical and social issues and ways to explore them further.

### Ethical challenge: social stratification of bioprinting

The promise or the hype of print your own organs is the most attractive solution to organ replacement avoiding sociocultural issues relating to possible breaches of interspecies and intraspecies integrity. For example, since the first human heart transplant narratives of some form of donor identity persisting and effecting the recipients, the regeneration of ‘self-organs’ will eliminate reported incidences of recipient changes in behaviour, identity and preference.^47^ Yet, the promise and hype is out of proportion to the possibility of realising 3D bioprinting and even in the possibility that 3D bioprinting was made a reality, it raises again the ethics of an increasing reliance on high sociotechnical solutions to middle-income country diseases. These are expensive technoscientific solutions likely to benefit only a few members of a particular subgroup (and brings to the fore of accessibility that needs addressing for regenerative medicine and tissue engineering). Therefore, 3D bioprinting is another solution that will not be a game-changer for everyone and certainly not for most in its immediate applications. Despite the promise of organs printed on demand for all, it is likely that the spectre of a ‘social stratification of biofabrication’ will emerge, with those who can afford to pay for their ‘own’ organs benefitting. A tiered system of therapeutic organ replacement is likely with those who can afford to pay for self-organs living longer; perhaps enjoying a significantly high quality of life avoiding the negative physical consequences of taking immune-suppressant drugs. While others will wait until a human organ donor becomes available and then have to undertake a punishing drug regime for the rest of their lives to prevent episodes of rejection of the transplanted organ. Others who cannot afford to pay then will make do with ‘second-hand’ organs from another living or deceased donor once available (as is the current system).

### Ethical challenge: managing expectations: avoiding the hype

It is important to note that while 2D bioprinting (tissues such as skin) and hollow tube printing (such as blood vessels and heart valves) are simpler, and therefore are more achievable on a short-term timeframe, hollow organs (such as the bladder) and solid organs (such as the liver and kidney) are far more complex and will require long-term development.^22^ Scientists are unable to put forward a concrete timeframe, due to the unpredictability of various challenges that need to be overcome. Indeed, it is a matter of some debate whether an organ can be successfully printed. Regarding organs one would have to print the organ and all the complex vasculature within it. As Mironov *et al* 48 suggest, ‘[A]chieving the desired level of cell density, effective vascularisation and accelerated tissue maturation are remaining challenges’. Problems also persist at the level of granularity. Indeed they have suggested that:

The only economic and reasonable way to commercialize organ-printing technology is to systematically employ scalable automated robotic technology and to build an integrated organ biofabrication line. It is not sufficient to develop just one robotic device—a bioprinter…[it] will require the development of series of integrated automated robotic devices, or an organ biofabrication line.^48^

### Ethical challenge: ethics of untested paradigms: living cells

3D bioprinting remains an untested clinical paradigm and is based on the use of living cells placed into a human body; there are risks including teratoma and cancer, dislodgement and migrations of implant. This is risky and potentially irreversible. Most studies showed short-term success; however, more long-term in vivo studies are required to show if side effects may emerge. Obviously, the need for cell source for bioprinting raises ethical issues around ESCs, in line with more common debates on their use. Despite promises of personalised medicine where adverse drug reactions can be tested by an ‘organ-on-a-chip’ technology, and therefore offer ‘cruelty-free drug testing’, how can medicine that is so highly personalised be then social stratified to the rest of the population? Who would be first in trial for example?

### Ethical challenge: ownership of printed bio-objects

Legal offerings suggest that bioprinting enters new territory distinct from previous legal regulation on medicine or on conventional 3D printing in relation to legal ideas of the body and the rights and responsibilities held by different groups.^9^ ^10^ ^12^ ^13^ Harbaugh^9^ focuses mainly on issues around ownership and the potential value of bioprinting products to different interested parties, including physicians, researchers and biotechnology companies. This is accompanied by a duty, however, to maintain the patients’ autonomy over their bodies. It is similarly suggested by Tran^12^ that benefits of bioprinting outweigh what he considers to be comparatively low risks, but that joint regulation between the medical and legal professions is needed to prevent new forms of exploitation such as a new black market in biofabricated organs.

The article by Li^10^ looks in further depth at potential intellectual property frameworks for bioprinting. An important distinction she raises relates to whether bioprinters should be categorised as machines used for a medical purpose, and thus a patentable entity, or non-patentable medical techniques involving direct printing onto or into the body, and thus not possible to be patented through the legal ‘medical treatment exception’. Similarly, products must pass the ‘morality test’ that it is morally acceptable to patent the item or process. These legal conditions in themselves raise pertinent ethical questions about the nature of bioprinted products—should they be regarded more as a type of (profitable) technology, or as a skill/treatment of the future? Organisations such as the United States Food and Drug Administration have also faced complexity when categorising bioprinted materials.^13^

Were the conditions for patenting of bioprinting to be satisfied, Li highlights potential dangers when it comes to the monopoly of particular scientific innovations stifling further innovation and also meaning that access to scientific benefits can be grossly unequal, which relates to this article's previously discussed fears of social stratification. However, others such as Varkey and Atala also highlight intellectual property provisions as able, from a scientific perspective, to protect the various steps of the bioprinting process.^13^ With many parallels to the current paper's later suggestion of the need for RRI, Li makes her own suggestion of a ‘portfolio approach’ to legal licensing in bioprinting.^10^ This places responsibility on companies to share benefits as well as emphasising the key role of publicly funded research.

Reflections are required on the kind of objects that could be created. What are bioprinted organs, how can we think about them and categorise them while they are crossing the conventional boundaries between life and non-life, for example, as bio-object^49^ or active matter (http://activemattersummit.com)? Questions arise about whether a refinement of current legislation or the introduction of new legislation, for example, how to deal with a hybrid bio-object? To whom do 3D bioprinted organs belong, and who has the right and/or opportunity to grow their own?

### Governance and regulations: responsible research and innovation

As demonstrated, social science and ethics might occupy a gap when it comes to issues around the development and production of 3D bioprinted organs. Wang *et al* 50 argue that:

[A]s a new topic of study, safety guidelines have not been firmly established in this field. The side effects of bioprinting have rarely been addressed, including questions such as biomaterials degradation and tissue integration, biocompatibility, and continuous tissue synthesis during material degradation.

Similarly, Guillemot *et al* 17 bring attention to data protection in the context of bioprinting. Therefore, we do not prescribe a research agenda for future work but modestly highlight the need for one. Furthermore, there is a variety of ways that governance of 3D bioprinting might be discussed. In conclusion, we highlight one such framework.

As an emerging field of study, the way in which science policy and research funding are stimulating biofabrication in general, and specific lines of research within bioprinting in particular is fundamental. Obviously, and because of its interdisciplinary nature, bioprinting requires the building of bridges on different levels.^51^ How are current and projected developments redrawing those disciplinary boundaries, in the lab between scientists with different backgrounds, and in terms of policy and funding? For example, in the British context, bioprinting research involves collaborations between different funding sources, cutting across the Biotechnology and Biological Sciences Research Council (BBSRC), Engineering and Physical Sciences Research Council (EPSRC), Medical Research Council (MRC) and National Centre for the Replacement Refinement and Reduction of Animals in Research (NC3Rs). An important question is how such collaboration for biofabrication is best shaped^52^? Moreover, and as bioprinted products do not fit current clinical trials governance,^53^ existing regulations need to be rethought and perhaps redesigned to guarantee the safety that Wang *et al* 50 and Guillemot *et al* 17 ask for.

### Towards a new framework to discuss ethics: from ethical legal social implications to RRI

While opening-up this debate through the outlining of relevant questions that need to be considered, we also would like to suggest a way forward in answering them. Similar to the scientific/technological shift—from regenerative medicine relying on animals to bioprinting, bringing new benefits and challenges—there is also a shift taking place in the framework in which reflections on new developments in the (bio)sciences are performed. While genomics came together with research on ethical, legal and social implications or aspects (ethical legal social implications (ELSI) and/or ethical legal social aspects research), there is now a trend towards more upstream ethical and social engagement through so-called RRI. This new oeuvre in science policy,^54^ which developed mainly in the context of nanotechnology and gains momentum within synthetic biology, seems to be a proper framework to tackle issues around emerging technologies on the boundary between life sciences and engineering and on existing approaches, such as constructive technology assessment. RRI is proposed as the ongoing process of aligning research and innovation to the values, needs and expectations of society, which requires that all actors, including civil society are responsive to each other and take shared responsibility for the processes and outcomes of research and innovation.^55^ By building trust between citizens, and public and private institutions, and by collectively assessing the risks as well as the way these risks should be managed, it is hoped that research outcomes will contribute useful, smart, inclusive and sustainable solutions to defined societal challenges.

The application of the RRI framework to emerging biotechnologies and objects simultaneously builds on and departs from the previous ELSI approach developed in the context of the Human Genome Project. It continues ELSI research through sustaining research into ethical, legal and social dimensions that emerge through novel developments in the biosciences. However, it importantly departs from the ELSI reactive stance—focussing on *implications* of scientific research—through the actual integration of social and ethical research in the scientific research practice. This concretely means that scientists and their social sciences and humanities counterparts are working together in research projects or centres to continuously interact and influence each other's thinking and the framing of the new technologies and their applications, while also connecting to societal actors and different users and publics.

The benefits of this integrative and collaborative approach can be illustrated through a recent paper which outlines an ELSI approach to bioprinting.^15^ In this paper, authors present various recommendations to discuss 3D bioprinting technologies so it can be effectively regulated while harvesting its benefits for public health. We certainly recognise the value of their suggestions to further ethical debate, yet it is important to note that their recommendations are reactive in the sense that they emphasise how ethical, legal and social aspects play an important role in the successful market translation of technologies (p. 19). Consequently, they do not envision the integration of ethicist and social scientist in scientific practice when the technologies are collaboratively imagined and potentially realised. However, and in light of our analysis that identifies a substantive gap between current practice and potential outcomes, we argue that the more proactive RRI approach may be a preferred alternative framework, as it brings ELSI into the laboratory and scientific conferences, contributing to its earliest development stages as well as to their translation.

RRI itself is still very much a concept in development and as such, we are pursuing collaboration between the scientists working in the realm of bioprinting and social scientists working on the emergence and ethics together of 3D bioprinting as exemplified by this article that is collectively authored.^56^

## Conclusions

The social and ethical challenges around 3D printing have received little commentary despite the schism between social promise and progress in technological terms. At first sight there seems less cause for angst as regeneration of human organs is less ethically fraught when compared with other technoscientific solutions such as animal sources. Moreover, the 3D bioprinting technology could potentially prove to be a game-changer in terms of how the human body is repaired or replaced. Nevertheless, there remain issues around general access and equity, biological and engineering responsibilities in terms of functionality matching with in vivo organs as well as the ethical governance of process, object and outcomes.[Supplementary-material SM1]

10.1136/medethics-2015-103347.supp1 supplement appendix
