# Supplementary material for: 3D bioprint me: a socioethical view of bioprinting human organs and tissues
Source: J Med Ethics. 2017 Mar 20;43(9):618–24. doi: 10.1136/medethics-2015-103347 (PMC5827711; doi:10.1136/medethics-2015-103347)

Appendix

Diagram 1: Literature search sources for social science literature on biofabrication undertaken in March-June 2016

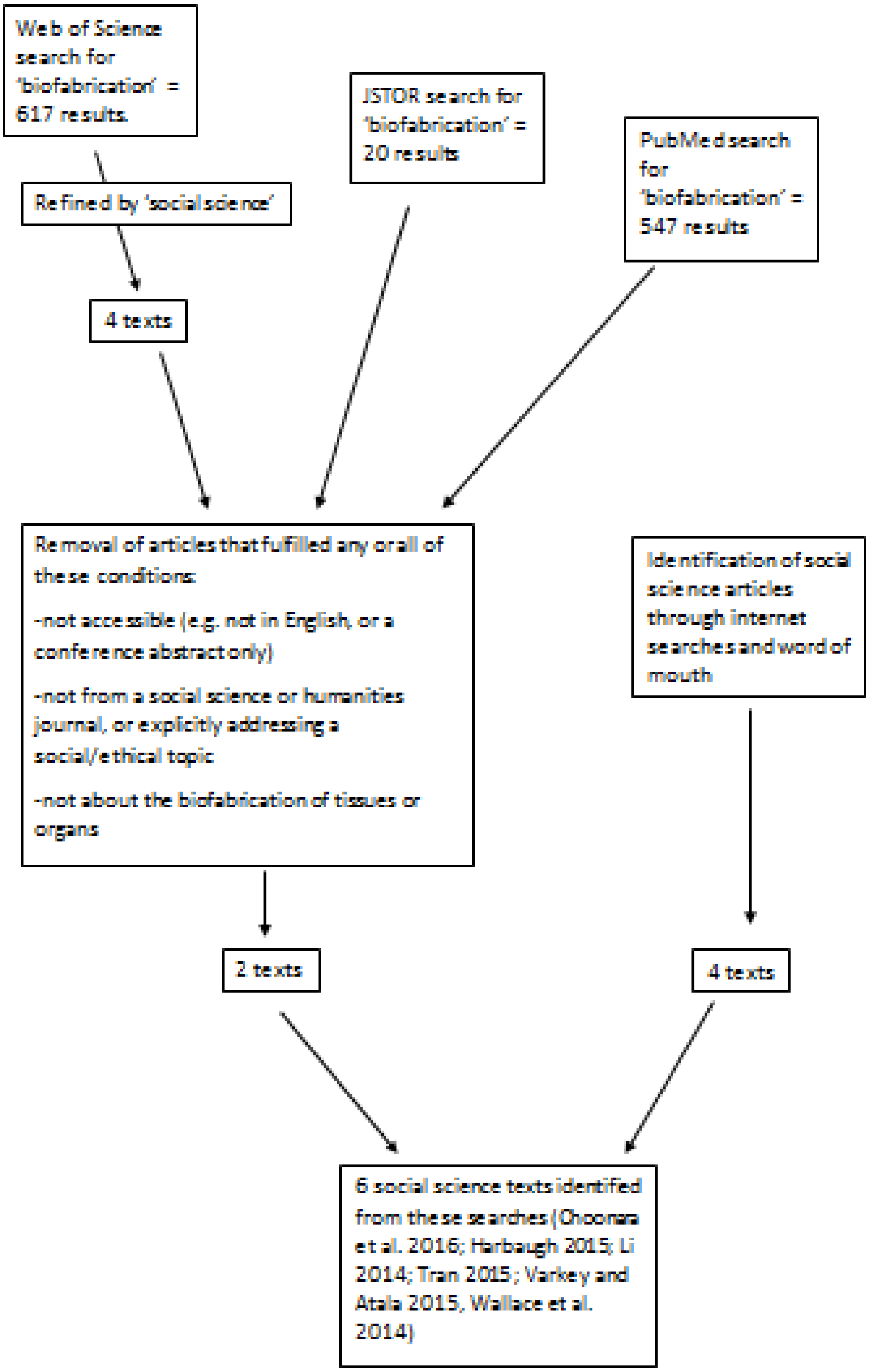

Supplement: supplement appendix [file medethics-2015-103347supp001.pdf]
